# Multispecies Bacterial Biofilms and Their Evaluation Using Bioreactors

**DOI:** 10.3390/foods12244495

**Published:** 2023-12-15

**Authors:** Grishma S. Prabhukhot, Charles D. Eggleton, Jitendra Patel

**Affiliations:** 1 Department of Mechanical Engineering, University of Maryland Baltimore County, Baltimore, MD 21250, USA; grish1@umbc.edu (G.S.P.); eggleton@umbc.edu (C.D.E.); 2 US Department of Agriculture, Agricultural Research Service, Environmental and Microbial Food Safety Laboratory, Beltsville, MD 20705, USA

**Keywords:** multispecies biofilm, bioreactors, foodborne pathogens, sanitary design

## Abstract

Pathogenic biofilm formation within food processing industries raises a serious public health and safety concern, and places burdens on the economy. Biofilm formation on equipment surfaces is a rather complex phenomenon, wherein multiple steps are involved in bacterial biofilm formation. In this review we discuss the stages of biofilm formation, the existing literature on the impact of surface properties and shear stress on biofilms, types of bioreactors, and antimicrobial coatings. The review underscores the significance of prioritizing biofilm prevention strategies as a first line of defense, followed by control measures. Utilizing specific biofilm eradication strategies as opposed to a uniform approach is crucial because biofilms exhibit different behavioral outcomes even amongst the same species when the environmental conditions change. This review is geared towards biofilm researchers and food safety experts, and seeks to derive insights into the scope of biofilm formation, prevention, and control. The use of suitable bioreactors is paramount to understanding the mechanisms of biofilm formation. The findings provide useful information to researchers involved in bioreactor selection for biofilm investigation, and food processors in surfaces with novel antimicrobial coatings, which provide minimal bacterial attachment.

## 1. Introduction

Biofilm formation in food processing industries can lead to health risks and financial losses for consumers, leading to unproductive resource consumption. Biofilms are well-organized microbial aggregates embedded in an extracellular polymeric matrix and adhere to biotic or abiotic surfaces [1,2,3]. Biofilms are generally defined as a cluster of microbes that thrive on biotic and abiotic systems and surfaces [4]. However, they are not simply a part of a “gathering”, but are instead very active, 3D, dynamic, and possess complex functionalities. Although the term “biofilm” itself does not have a widely accepted definition, according to Lewandowski et al. [3], it can be defined as follows: “a biofilm is considered to be an aggregate of microorganisms embedded in a matrix composed of microbially produced extracellular polymeric substances (EPS) and attached to a surface”. As a result of evolution, by exhibiting homeostatic responses and gene regulations, the biofilms help microorganisms to proliferate in extreme environments [5].

### 1.1. Stages of Biofilm Formation

**Figure 1:**
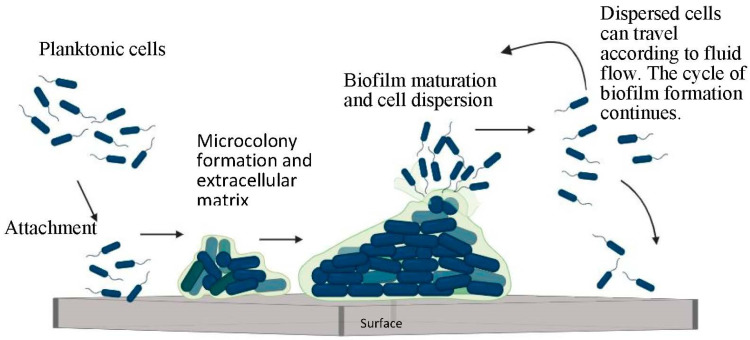
Schematic diagram of biofilm formation.

Microorganisms undertake multiple steps to form biofilms, such as initial attachment, microcolony formation, maturation, and dispersion [1], as shown in Figure 1. The initial contact of the bacteria with a substrate is reversible. Multiple physical and chemical forces, such as electrostatic and cohesive forces, play a vital role at this stage. The appendage structures, like the fimbria and pili, present on the cell membrane strengthen the bacteria–surface attachment. After getting attached to the surface, the bacteria start to divide and multiply. During this developmental stage, extracellular matrix formation takes place [6]. This matrix encompasses an extracellular polysaccharide substance (EPS) and structural proteins, among other components, which keep the colony safe from external variables, and this phase is called “irreversible attachment” [1]. Microcolonies form during this irreversible stage of attachment. Microcolony formation gives rise to cell–cell communication (quorum sensing) [6,7]. Bacteria in the microcolonies use “quorum sensing” to exchange information among themselves, and this enables the EPS to modify in response to and withstand any changes in the environment (such as pH, temperature, pressure, concentration and shear stresses). Based on this communication, the bacterial behavior can become more cooperative, competitive, or mutually beneficial [8]. The formation of small channels within the microcolonies facilitates nutrient distribution among the bacteria in biofilms [5]. The last stage of biofilm formation is characterized by the natural dispersion of bacteria from existing microcolonies. When the bacteria become naturally detached from their biofilm, either as single cells or as a cluster, they seek to colonize their environment, thereby acquiring new sites in the system, and this gives rise to an even further increase in biofilm formation [9].

### 1.2. Social Dynamics: Cooperative and Competitive Interactions in Biofilm Consortia

As mentioned earlier, biofilms have been involved in the evolutionary biology of bacteria competing for nutrients, co-evolving with other organisms via either inter-dependency or by opportunistically exploiting other bacterial species, allowing them to evolve and sustain in nature. When multiple bacteria exist in a consortium and form a biofilm, they engage in social behaviors such as quorum sensing, and metabolic competitive or cooperative interactions [10]. By taking the form of such a group, these bacteria make multiple survival decisions based on external stressors such as UV light, temperature, pH, pressure, and antimicrobial agents. The definitions of these behaviors are often confounding because an advantageous behavior for one species can be detrimental for the other; so, it can be confusing whether to define a specific behavior as cooperative, because one of the species is flourishing, or as competitive (antagonistic), because the other species is on the verge of being removed from that environment. Cooperative interactions can lead to the formation of desirable spatial organizations that enhance the supply of nutrients throughout biofilms [11]. Besides this, coaggregation plays a vital role in biofilm formation via the involvement of specific surface structure such as pili and flagella. Cooperative interactions can also protect the biofilms from antimicrobial agents by producing strong EPS. A study by Lee et al. [12] discovered this behavior when using *Pseudomonas aeruginosa*, *Pseudomonas protegens*, and *Klebsiella pneumoniae* to form multi-species biofilms. The spatial structures of the multi-species biofilms were different, but they were also more resistant to antimicrobials than single-species biofilms. More importantly, Bridier et al. [13] reported that *Pseudomonas* biofilms could alter their exopolymer matrix to increase their resistance against antimicrobials, such as chlorhexidine, benzalkonium chloride, or triclosan. While spatial distribution is an inherent morphological attribute of biofilms, it is strongly influenced by chemical stressors.

Competitive interactions in multi-species generally arise due to the limited availability of nutrients [14]. It can be debated as to whether biofilms prefer competitive or cooperative behaviors. Nonetheless, these behaviors lead to the expression of virulence factors, antibacterial proteins, and gene expressions. For example, competition can alter the quorum-sensing response of the bacterial species in biofilms [15]. In multi-species biofilms of *P. aeruginosa* and *E. coli*, the sustenance of *E. coli* is determined by the production of indole by the *E. coli* bacteria. However, when the *E. coli* mutates, its mutants may not synthesize indole, which should result in the abundant growth of *P. aeruginosa* with subsequent reduction in *E. coli* populations. However, indole blocks the toxins and quorum sensing phenotypes of *P. aeruginosa,* thus benefitting *E. coli* survival [16]. Table 1 summarizes some of the recent studies related to the social behavior of biofilms.

### 1.3. Influence of Fluid Dynamics on Biofilm Formation

Irrespective of the bacterial strains, some of the common factors such as equipment surfaces, environmental conditions, and hydrodynamic shear forces involved in the movement of foods at the food processing plants, as well as water/chemical use during cleaning and sanitation could influence biofilm formation. Biofilms show a very peculiar response to these external factors, including temperature, humidity, pH, and flow conditions such as static flow, rotary flow and circulatory flows, as well as the sanitation regime. Under laminar flow, the biofilms take the form of mound-shaped microcolonies, whereas under turbulent flows, their structure is more filamentous and somewhat streamlined, with a defined “head” and “tail” [25]. Moreira et al. [26] reported the effects of shear stress on biofilm formation. In their study, an increase in *E. coli* biofilm formation was observed at the lowest shear stress of 0.183 Pa compared to a higher shear stress of 0.365 Pa. However, contrary results regarding the biofilm and shear stress relationship were also reported [27,28]. This study compared shear stress values of 0.007 (laminar), 0.02 (transition) and 0.07 N/m^2^ (turbulent), and observed higher biofilm development under turbulent flow. These observations suggest that the formation of biofilms under laminar or turbulent flows may also depend on the fluid dynamics involved in the biofilm equipment used to evaluate the effects of the hydrodynamic shear stresses applied.

Fluid flow conditions are commonly classified into laminar, transition or turbulent regions using the Reynolds number [29], which is a dimensionless number that was developed in 1883 by Osborne Reynold [30]. For a smooth circular pipe, the flow is considered to be laminar if Re < 2100, transient if 2100 < Re < 10,000, and fully turbulent if Re > 10,000 [29]. The exact values used for classifying the flow depend on the conduit’s geometry, the surface roughness, the fluid viscosity and other flow parameters. Typically, laminar flows are classified by a constant local velocity with respect to time, transition flows with intermittent bursts of turbulence or mixtures of flow streams, and turbulent flows with random fluctuations among the flow streams.

Fysun et al. [31] reported the effects of flow hydrodynamics on biofilm development using *Pseudomonas fragi* and pasteurized milk containing *Streptococcus* spp., *Bacillus* spp., and *Micrococcus* spp. They confirmed that laminar flow contributed more significantly to biomass (mg/cm^2^) formation than turbulent flow. The authors noted that in the laminar flow region, biofilm development was primarily influenced by mass transport, and in the turbulent flow region, it was heavily influenced by shear stress. Furthermore, the flow conditions in the bioreactor itself were frequently simplified. However, certain details of the study, such as the locations of the coupons, the attachment mechanisms of the coupon to the pipe, the length of the pipe, the bacteria’s residence time, as well as results of flow analysis, were unavailable. It is crucial to know the coupon location, the coupon size, and the effects of coupon placement on flow disturbances (flushed/non-flushed), if any. Such knowledge will help in avoiding flow separations, bubble formations and local eddies that might occur at the site [32]. Also, the strategic positioning of the coupons is required to ensure that the samples are collected from different locations of the tube, instead of placing them in the same positions around the circumference [33]. It is equally important to investigate whether the probability of biofilm formation is uniform throughout the flow channel and to ensure the recirculation of flow in the tubes [34]. The presence of gradients can induce variability in the environment of biofilm formation [34,35], which requires investigation, specifically in the flow path and nutrient reservoir.

Similarly, a study by Oder et al. [36] highlighted the behavior of biofilm formation using *E. coli* under different hydrodynamic shear stresses (static, laminar and turbulent). The study traced the advances in cell multiplication over periods of 24, 48, and 72 h. The initial inoculum was 7 log CFU/mL, and nutrient broth was used as a growth medium with no subsequent addition of nutrients to the system. During the first 24 h, higher *E. coli* populations were recovered from biofilms under turbulent flow than static and laminar flow conditions. However, this trend was reversed after 72 h of exposure to the flow conditions, with the lowest *E. coli* populations seen under turbulent flows. The lower cell multiplication rate under turbulent flows was attributed to the numerous bacterial presence, leading to increased competition for nutrient consumption. Overall, the biomass of biofilms under laminar flow increased, whereas under turbulent flow, it decreased over time on the stainless-steel surfaces.

Another significant aspect of flow hydrodynamics affecting biofilm development was investigated by Lemos et al. [37] using *Bacillus cereus* for biofilm formation under turbulent flow conditions. The shear stresses used for this study were approximately 0.02, 0.12, and 0.17 Pa, and the Reynolds numbers of agitation were 1000, 3200, and 4000, respectively, under the turbulent flow regime. In this study, the biomass content was the lowest for biofilms formed under low shear stress. Similarly, cell density and the extracellular polysaccharide content were also lower under low shear stress. Further, the biofilm density increased, while its thickness decreased, under turbulent flow. Biofilms subjected to turbulent flow not only showed an increased cell density (bacterial population per cm^2^), but also showed an increased volumetric density (mg/cm^3^) and biofilm mass (mg/cm^2^). Vieira [38] investigated the effects of laminar and turbulent flow at Re values of 2000 and 5200, respectively, on *Pseudomonas fluorescens* biofilm formation. The findings of this study resonate with the findings derived by Lemos et al. [37], who reported that the biofilms showed a higher cellular density and increased mass per cm^2^ under turbulent flows. The use of a rotating cylindrical reactor (RCR) in simulating the flows in elbows or pockets used in a specific industry might require the correlation of the flow specifics of these devices. Pipe fittings such as elbows or dead ends are involved in the back-mixing of the fluid. Rotating annular cylinders might induce the development of vortices on their surfaces. Also, the presence of multiple cylinders in the RCR might result in vortices interfering in the section between the two cylinders, leading to laminar flow with eddies or turbulence, especially when the desired flow is laminar. This specific area of correlation between the bioreactors and the pipe fittings’ actual dimensions has been overlooked in studies [37].

Several authors have recognized the effects of different flow conditions on biofilm formation. One of the flow patterns involves the “mixing” of different liquids or semi-solid foods, which is a ubiquitous operation in the food processing industry. To promote fluid motion in a homogenized mixture of bacterial cultures, the selection of a suitable size and type of impeller, as well as the blade number and blade curvature, are all essential. Usually, two-blade impellers are used for the smooth mixing of fluid in the industrial setting [39]. At the bottom of the tank, eddies may form where the impeller blade almost touches the surface but with no friction, and this could reduce the flow velocity near the bottom of the tank. Similar observations were also made by Ismadi et al. [40]. According to their study, a region with higher shear stress developed at the base of the spinner flask bioreactor, and a recirculation structure formed in the bioreactor. However, the impeller’s position did not directly impact the bioreactor’s shear stress.

In continuous-stirring tank reactors (CSTRs), different impellers can be used based on various factors, such as fluid viscosity and desired flow patterns. A unique feature of CSTRs is that the fluid flows in and out of the reactor continuously. Therefore, the environment inside the bioreactor can become unstable. According to Kadic et al. [41], maintaining residence time of bacteria is important in order to achieve a uniform environment throughout the batch process. Reactor geometry and material “in flow–out flow” play essential roles in maintaining the residence time. Stirred tank reactors (STRs) can display significant levels of back-mixing of fluids. If the eddies generated in the STRs are the same size as the bacterial cells, then the reactor’s hydrodynamics can damage the bacterial cell wall. Thus, STRs are suitable for use with shear-resistive microorganisms. Furthermore, Csapai et al. [42] compared the effects of flow and electric field on biofilm formation in a microfluidic device, and found that biofilms grown under static conditions were well defined compared to biofilms subjected to the low flow rate of 0.1 mL/min (Reynold’s number of 1.49 and flow velocity of 0.69 mm/s). The fluid flow conditions and flow parameters used in some biofilm studies are summarized in Table 2.

### 1.4. Influence of Surface Material on Biofilm Formation

Existing studies in the broader literature offer a wide-ranging perspective on the effects of abiotic surfaces on microbial attachment [53,54,55]. Some of the factors reasoned to impact biofilm formation include surface roughness, hydrophobicity, material properties, surface charge and surface finish. De-la-Pinta et al. [56] studied the effects of polycarbonate, silicone, titanium, borosilicate, and Teflon on the biofilm-forming abilities of *E. coli*, *P. aeruginosa*, *S. epidermidis*, and a *C. albicans*. In their study, the surface materials were machine-finished to alter their surface roughness using silicon carbide grinding papers with grit numbers 320, 800, 1200, and 4000. They observed that modifying the surface roughness affected the wettability of the surface. Surface hydrophobicity is not an inherent physical property of a material, and it can be altered via changes in surface roughness. Teflon, silicone, and polycarbonates are classified as more hydrophobic, and overall, *E. coli* biofilm formation was higher on these materials. This is a noteworthy observation because of the conventional and acceptable use of Teflon on the coatings of cookware. In general, if the diameters of the bacteria were greater than the degree of surface roughness of the material, staphylococcal biofilms were found to be sparse. This observation was contradicted when the untreated surface was compared with a surface polished with a 4000-grit smoother. The results of this study imply that the degree of symmetry of surface heights on the mean plane (Ssk) might play an essential role in determining the degree of microbial attachment to the surface. According to this study, the prevalence of peaks on a surface promoted microbial attachment irrespective of the surface roughness. Goulter-Thorsen et al. [57] indicated that *E. coli* O157 cells attached in higher numbers to the smoother stainless steel surface (#8 finish) when compared to the rough surface (#2 finish). While De-la-Pinta et al. [56] observed that modifying the surface roughness affected the contact angle, Goulter-Thorsen et al. [57] did not see a significant difference in the contact areas of SS with different surface finishes. On the contrary, other studies have suggested that neither surface hydrophobicity nor surface irregularities influenced bacterial attachment [58].

Cheng et al. [59] utilized a fermenter bioreactor to cultivate *Acetobacter xylinum* biofilms on plastic composite support materials used for cellulose production. The cellulose obtained from the support material in the bioreactor exhibited superior mechanical strength when compared to cellulose produced in an agitated culture. Roveto et al. [60] evaluated the impacts of nitrifying biofilms on PDMS-Methyl, PDMS-Ester, and PDMS-Amine surfaces in annular batch-type bioreactors using *Nitrosomonas* and *Nitrospira* bacteria. In their study, the higher surface energy of the amine surface resulted in the increased adhesion of these bacteria compared to the degree of attachment to hydroxyl and methyl surfaces. In contrast, the uncharged hydrophilic surfaces lacked a diverse range of species in their biofilms, and were primarily dominated by Acinetobacter biofilms. Vongkampang et al. [61] investigated biofilm formation using *Caldicellulosiruptor kronotskyensis* and *Caldicellulosiruptor owensensis* in a continuous flow process applied to jacketed glass using acrylic fibers and chitosan. The combination of acrylic fibers and chitosan contributed to stable biofilm formation due to the production of tāpirin proteins by the bacteria, which facilitated greater attachment to lignocellulosic substrates. Recently, Yang et al. [62] investigated the biofilm formation of *Salmonella Typhimurium* and *E. coli* O157:H7 in static bioreactors containing stainless-steel coupons. The degree of attachment of these pathogens varied with the bacteria used, and they attached more efficiently to surfaces compared to other bacteria used in meat processing.

**Figure 2:**
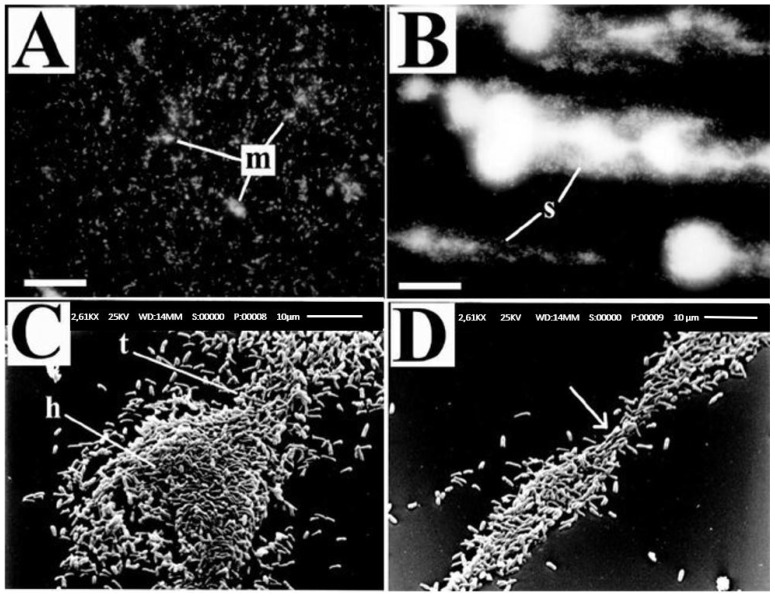
*P. aeruginosa* PANO67 biofilm grown under (**A**) laminar flow—small mound-shaped microcolonies (m) and single cells; (**B**) turbulent flow—filamentous streamers (s); (**C**,**D**) “head” and “tail” of a *P. aeruginosa* PANO67 biofilm grown under turbulent flow conditions. The direction of flow is from the bottom left to the upper right (adapted from [25]).

Stoodley et al. [25] have reported on the effects of hydrodynamic forces on bacterial biofilm detachment. They studied the mechanical properties of *P. aeruginosa* biofilms formed using an in-vitro flow cell under various hydrodynamic conditions, and subsequent biofilm deformation and detachment. The two sets of experiments undertaken focused on biofilm formation under laminar flow with a Reynolds number of 8, 0.002 m/s flow velocity, and 0.03 N/m^2^ shear stress, and under turbulent flow with a Reynolds number of 3600, 1 m/s flow velocity, and 5.09 N/m^2^ shear stress. Even though the authors intended to study deformation under shearing forces, the biofilms’ irregular structures gave rise to complex local flow patterns. The forces acting on the biofilms were a combination of shear and normal forces. Interestingly, the biofilms formed under laminar flow showed isotropic surface patterns compared to the biofilms formed under turbulent flow (Figure 2). The turbulent flow biofilms had a structure with a pronounced “head” and “tail”. Biofilms formed under turbulent flows featured filamentous streamers oriented in the downstream direction. These biofilms showed “necking” (a peculiar phenomenon specific to ductile materials) during their failure under externally applied shear stress [25]. Most importantly, this research deduced a relationship between the shear force used for biofilm growth and the applied shear force required to detach the biofilm. According to this study, the cells started to detach when the applied external shear stress increased to approximately twice the shear stress under which the biofilm formation occurred. Moreover, they observed that the *P. aeruginosa* behaved like a viscoelastic fluid when attached to the substrate.

Another significant study on biofilm detachment undertaken by Stewart [63] mentioned that the biofilms would detach if the applied external stress exceeded its failure strength (the applied force per unit area required for the biofilm to break). Also, this author recognized that biofilms are inherently heterogeneous. Paul et al. [64] provided a rare insight into the biofilm detachment mechanism, showing that the biofilm resists detachment at increasing shear stresses when undergoing compaction caused by the applied external forces. By conducting a 2D image analysis, they also observed a “basal layer”, where the biofilm was more cohesive and denser than in the outer layers.

Biofilms exist on biotic and abiotic surfaces in nature. In industrial settings, microbial biofilms are found on various surfaces in food processing plants, such as on dispensing tubing, heat exchangers, silos, pipelines, conveyor belts, tables, pallet jacks, walls, water pump exteriors, employees’ gloves, contact surfaces, as well as packing materials [31,65]. Generally, equipment employed in the food industry is made of materials such as stainless-steel, Teflon, glass, silicone, polycarbonate and synthetic rubber. Previous studies have shown that the formation of biofilms on these surfaces depends on factors including surface properties, such as hydrophobicity and topography, fluid flow conditions, as well as the physicochemical properties of the bacteria, such as the activation of genetic cascades, the age of bacteria and the presence of exopolymeric substances [66,67]. The influence of surface properties, such as hydrophobicity and surface topography, on the biofilm formation capacity of *E. coli* is not well understood. While some studies have concluded that abiotic surface roughness determines the outcome of biofilm development [57,68], other studies have found that a substrate’s hydrophobicity plays a vital role in biofilm formation [56]. Thus, biofilms’ abundance on a given abiotic surface might depend on multiple factors, such as the substrates present and the physicochemical properties of the bacteria. Further research is needed to understand the fundamental factors that may determine harmful biofilm formation in food processing industries. Investigations seeking new biofilm removal techniques should replicate the bacterial strains, substrate materials, flow hydrodynamics and overall environment encountered in the food processing context to ensure effective intervention. To ensure these experimental parameters, it is imperative to select bioreactors to be used in biofilm studies that simulate industrial or processing conditions. Bioreactors offer a controlled environment that helps investigators to study the multiple variables affecting biofilm formation. These variables include the surface characteristics of materials, nutrient composition and availability, bacterial characteristics and their interactions, fluid flow conditions, and environmental conditions such as temperature, pH, and OR potential. Further, biofilm studies can be undertaken to develop subsequent intervention strategies to be used in the removal of biofilms from surfaces. Multiple types of bioreactors that are available for investigation in terms of operational mode, with static or dynamic principles, are discussed here.

## 2. Types of Bioreactors

To understand biofilm formation and develop preventative and control mechanisms for their removal, researchers have been diligently engaged for decades in developing suitable bioreactors [69,70,71]. Bioreactors can be used by researchers to develop and grow biofilms, control their growth, improve existing preventative strategies, and develop new interventions [72,73,74]. Previous and ongoing developments made in bioreactor design facilitate the study of various parameters, such as the surface, temperature, humidity, nutrient conditions, use of sanitizers, and the physiochemical and biological properties of bacteria, individually as well as in controlled integrated configurations. The selection of suitable bioreactors can impact the type of data generated by the bioprocesses, thereby influencing the outputs. As a result, the selection of bioreactors suitable for research is a crucial step in ensuring the reliability of the output, thereby leading to the development of accurate solutions.

**Figure 3:**
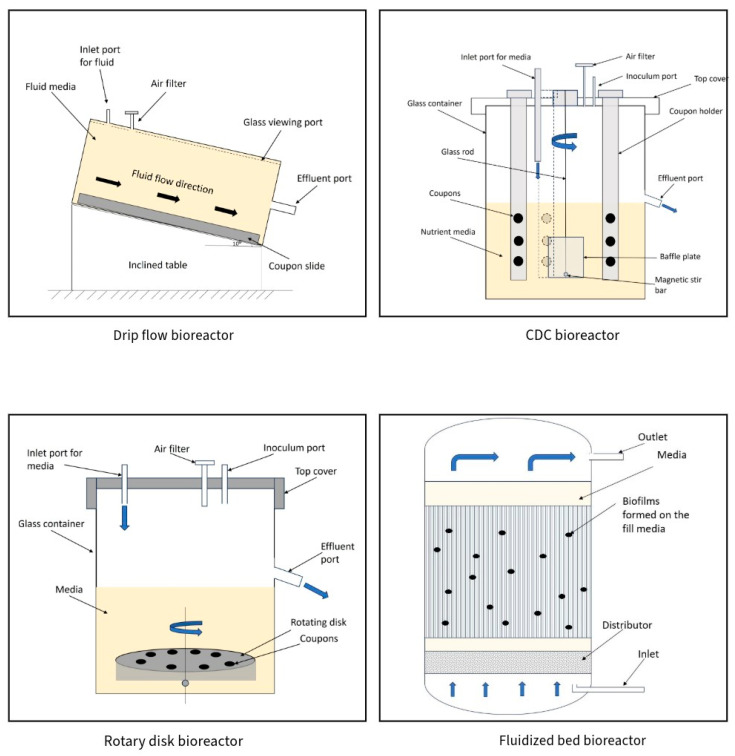
Schematic diagram of Drip flow bioreactor [91], CDC bioreactor [89], Rotary disk bioreactor [33], Fluidized bed bioreactor [94].

Bioreactors can be broadly classified according to their design principles, operation modes, size or scale of operation and environmental characteristics. Based on their design principles, bioreactors can be classified broadly as stirred-tank, air-lift and fluidized bed, and according to their operation modes as batch, fed-batch/semi-continuous, or continuous. The different types of bioreactors used in biofilm research are summarized in Table 3. Figure 3 shows a schematic diagram of the main bioreactors used in biofilm studies.

**Table 3:** Different types of bioreactors used in biofilm formation.

Classification	Bioreactors	Microorganisms	Study	References
Operation mode	Batch process	*Lactobacillus helvetics*	Biomass assessed for antimicrobial and probiotic properties	[75]
		*Bacillus* sp., *Lysinibacillus* sp., *Kerstesia* sp.	Wastewater treatment evaluation with decolorization/removal of Amaranth dye	[76]
	Fed-batch process	*Lactobacillus casei*	Evaluation of use of plastic-composite supports in fermentation; periodical spike to maintain ~8% glucose in a reactor	[77]
		*Bacillus subtilis natto*	Biofilm formation in glycerol and glucose-based media; bioreactor cycle every 12 h for Vitamin K extraction	[78]
	Continuous flow process	*Cronobacter*, *Listeria monocytogenes*, *Salmonella* and *S. aureus*	Multispecies biofilm formation in CDC reactor under turbulent flows to mimic dairy processing	[79]
		*Streptomyces* sp.	*Streptomyces* biofilms used for the removal of insecticides on polyurethane foam pieces	[80]
Static bioreactors	Lab equipment	Poultry slaughterhouse wastewater isolates (*Comamonas* sp.)	Bioflocculants were produced by optimizing conditions inside conical flask bioreactors using *Comamonas* sp. bacterial biofilms	[81]
		*Enterobacter cloacae*, *Klebsiella oxytoca*, *Serratia odorifera*, and *Saccharomyces cerevisiae* *Salmonella* isolates from swine *Salmonella* isolates of produce and poultry origin	Biofilm formation on moving bed media for the removal of mercury from wastewater Efficacy of natural antimicrobials in biofilm removal Comparative evaluation of bacterial sources in the context of biofilm formation	[82,83,84]
	Scaffolds	*S. aureus*, *E. coli*, and *P. aeruginosa*	Biofilm formation in clinical and food industries using Ɛ-caprolactone scaffold and curcumin nanofibers	[85]
		*Lactiplantibacillus plantarum*	Biofilm formation on electrospun ethyl cellulose nanofiber scaffolds to improve self-resistance of probiotics during production	[86]
	Microfluidic devices	*Enterococcus faecalis*, *S. aureus*, *Klebsiella pneumoniae* and *P. aeruginosa*	Evaluation of 3D-printed polylactic acid surfaces to construct a microfluidic device and its suitability in biofilm formation studies	[87]
		*P. aeruginosa*	Biofilm formation in microfluidic channels under different oxygen availability conditions	[88]
Dynamic bioreactors	Stir-tank	Shigha-toxigenic *E. coli*, *L. monocytogenes*	Efficacy of peptides used in removal of pathogenic biofilms	[89]
		*Xylaria karyophthora*, *Clostridium aceticum*, *S. aureus*	Inhibition of *Candida albicans* and *Staphylococcus aureus* biofilms using cytochalasins from *Xylaria karyophthora*	[90]
	Drip flow	*S. aureus* and *P. aeruginosa*	Mixed-species biofilm formation for evaluation of an anti-biofilm treatment	[91]
		*T. reesei* and *T. harzianum*	Adhesion of fungal biofilms on Viton rubber, stainless steel, PTFE, silicone rubber and glass	[92]
	Fluidized bed biofilm	*Nitrospira*, *Nitrobacter*	Carbonaceous oxidization and nitrification of wastewater with biofilm	[93]
		*Comamonas*, *Thiobacillus*, *Pseudomonas*, *Thauera*, *Nitrospira*	Multispecies biofilms used for the removal of chemical oxygen demand and ammonia nitrogen	[94]
	Modified Robbins Device	*Staphylococcus epidermidis*	Adhesion of *S. epidermides* to glass, siliconized glass, plasma-conditioned glass, titanium, stainless-steel, and Teflon	[95]
		*Candida albicans* and *S. aureus*	Evaluation of disinfectants used for biofilm removal on oral medical devices	[96]
	Flow chamber	Multiple oral commensal and pathogenic bacteria	Oral multispecies biofilm evaluation used in BHI/vitamin K medium	[97]
		*E. coli*	Biofilm formation on oral implant materials: glass and implant steel	[98]
	Rotating disk type	*Blakeslea trispora*	*B. trispora* biofilms for carotene production in fermentation system	[99]
		*Shewanella colwelliana*	Effects of surfaces on *S. colvelliana* biofilms and in melanin production	[100]

### 2.1. Classification Based on Bioreactor Operation Mode:

#### 2.1.1. Batch Process Reactor

In this type of bioreactor, all the products, such as nutrients, microorganisms, and other materials required for the process, are added, and the reactor is run uninterrupted until the available nutrients are depleted; the materials of interest are then recovered after the end of the operation cycle. A recent study by Bodean et al. [101] utilized a batch process to study the activities of herbicides and fertilizers on biofilms. *Cyanobacterial* biofilms were successfully recovered after the batch process for further qualitative and quantitative analysis. According to Mitra et al. [102], batch reactors require sophisticated control algorithms, since the feed is supplied only once, and changes that occur inside the bioreactor could challenge the reproducibility of the experiment. Along with the fact that the outputs are obtained at the end of the process, these are the major drawbacks of this type of bioreactor. Since the control of nutrients is relatively difficult to ensure in a batch reactor, there is a preference among researchers and industry practitioners to utilize fed-batch bioreactors.

#### 2.1.2. Fed-Batch Process Bioreactor

A fed-batch bioreactor can be employed to alter the nutrient concentration during the process, which is a capacity lacking in batch-type bioreactors. As the name suggests, the nutrients are “fed” intermittently to maintain the concentration. This type of bioreactor could also be used when the end goal is to not only derive the output from a consistent nutrient media, but also when modifications in the nutrient media are required. Unlike batch and continuous bioreactors, the total mass content in this type of bioreactor changes during the process. According to Yamuna Rani et al. [103], fed-batch bioreactors require users to develop protocols focusing on the control parameters using two approaches: a physiological model, and a dynamic optimization approach. A physiological model employs a specific parameter, such as the constant maintenance of the nutrient concentration based on conceptual analyses, which would not involve any mathematical modeling. On the other hand, the dynamic optimization approach would focus on the set-point tracking of parameters (such as pH, temperature) at regular intervals based on mathematical modeling. Germec et al. [104] used a fed-batch process to form *Aspergillus sojae* biofilms for β-mannanase fermentation. They stated a preference for this process compared to the batch-based one, as the latter displayed drawbacks such as more time required for cleaning and sterilization, the low resistance of microorganisms to shear force, and the lack of a re-inoculation capacity when required.

#### 2.1.3. Continuous Flow Process

In the continuous flow operation mode, fresh medium is continuously added to the reactor, and the effluent is discarded at the same rate at which the fresh media is added. Trappetti et al. [105] suggested continuous flow bioreactors as the most suitable for studying the mechanisms and growth of mature biofilms; specifically, they can help with developing an understanding of the spatial arrangements of the structure and the EPS of a biofilm. Continuous flow processes allow users to collect samples at different time points, thereby extending the productivity of the study, and due to the addition of fresh nutrients, the overall issue related to heat generation in the bioreactor can be eliminated, with the enabling of better temperature control. Nevertheless, this system is prone to an increased risk of contamination due to the frequent sampling required during the process. The most common types of bioreactors that utilize this operating mode include plug flow reactors and continuous stir tank reactors.

### 2.2. Classification Based on Working Principles

Reactors can be broadly classified into two types based on their working principle: static bioreactors and dynamic bioreactors. This broad classification can help us with the primary steps when selecting a suitable bioreactor for carrying out a desired process. The classical 96-well plate biofilm assay [33,106] is considered a form of static bioreactor. On the other hand, dynamic reactors require additional machinery to generate fluid motion in their container. Dynamic reactors are commonly found in food processing environments in the form of industrial mixers, dough-making machines, blenders, etc.

Stirred tank reactors are a commonly encountered type of dynamic reactor, which can be further classified by their end-use and basic design parameters. These reactors are characterized by the presence of ports at the top, as well as a stirrer or baffle plate. Impellers for the stirrer can be designed as per their compatibility with the fluids used. Winterbottom et al. [107] has explained in detail the different types of impeller designs available, such as wide-radius agitators and marine propeller-type agitators. These reactors are relatively more easy to design and model mathematically due to the fact that all the control parameters are contained in an enclosed unit during the reaction process. The mass balance of these reactors in batch mode has been explained previously [108].

### 2.3. Classification Based on Scale:

Biofilm bioreactors can also be classified based on the scale of application into either industrial or laboratory research-based. Laboratory-scale bioreactors include microtiter plates, agar plates, CDC bioreactors, drip-flow bioreactors, Bio-inLine^®^ (BioSurface Technologies, Bozeman, MT, USA) bioreactors, rotating cylinder annular bioreactors, constant depth film fermenters, and related modifications [33]. Microtiter plates and agar plates are not specifically designed for as bioreactors; however, their wells act as vessels for conducting controlled experiments with the minimal use of resources and preliminary research. CDC bioreactors and the other aforementioned reactors are specialized equipment that has been designed to conduct lab-based biofilm research to study specific applications. According to Goeres et al. [33], CDC bioreactors and annular bioreactors are commonly used to study dental biofilms, and are also used in biofilm removal studies as well as studies on biofilms used in food processing under high-shear conditions. Drip flow bioreactors are also used to conduct studies related to dental biofilms, biofilm control, and other applications similar to those of CDC and annular bioreactors; however, these studies are commonly conducted under low-shear environments. Although different lab–desk bioreactors can be modified and used for similar applications, their outputs or results might not be comparable due to the fluid dynamics and bioreactor design conditions [109,110].

## 3. Sanitary Design in Food Processing

In the realm of biofilm management, while the investigation of effective control measures remains imperative, it is of paramount importance to prioritize research efforts in the direction of preventative strategies [111]. Preventative strategies rely heavily on the design principles employed when constructing food contact surfaces and other surfaces present in processing facilities [112,113]. By directing our focus towards preemptive measures that can be taken in the area of equipment design, we can aid in mitigating biofilm formation. According to Moerman et al. [114], food safety legislations demand that processing equipment meets sanitary design standards, the result of which is minimized food contamination risk. However, it is necessary to underscore that hygienic design encompasses a broader spectrum of manufacturing practices that are always evolving. This diverse approach ranges from considering the capacity to clean the surface, the choice of mechanical fittings, the weldability, the radius of filets and corners, the surface skewness and kurtosis, the ease of assembly, etc. As these factors are also interrelated, much attention should also be paid to meeting the hygienic and sanitary design conditions, especially when these different criteria can interfere with each other.

### Surface Coating to Prevent Biofilm Formation

To control biofilm formation and facilitate their removal, as an alternative to using chemical agents known for their environmental effects and occupational hazards, the development of antimicrobial coatings is compelling. Antimicrobial coatings can be broadly classified into four categories: release-based, contact-based, repulsion-based and superhydrophobic effects-based [115].

Release-based coatings function by releasing controlled amounts of antimicrobials from the polymer matrix to inhibit the proliferation of bacterial cells. Antibacterial agents such as quaternary ammonium compounds, heavy metals, aldehydes, essential oils, alcohols, and halogens can be used as antimicrobial agents to impregnate the coating matrix. Recently, Regulski et al. [91] reported the efficacy of using silver as an antimicrobial substance in various dressings, such as dressings with a nanocrystalline coating of silver (Nano Ag) on the wound, silver-impregnated CMC-1.2% Ag dressings, and Poly-Sheet Metallic Ag and Polyurethane foam absorbent dressings containing silver salt against *S. aureus* and *P. aeruginosa* mixed-species biofilms. The populations of *S. aureus* recovered from membranes inoculated with mixed-species biofilms have shown significant reductions when Nano Ag was used (3.42 log CFU reduction), and significant reductions in *P. aeruginosa* populations (4.57 log CFU reduction) have been achieved with Poly-Sheet Metallic Ag wound dressings compared to untreated controls. The antimicrobial efficacy of silver nanoparticles-coated surfaces against *Streptococus mutans* biofilms was evaluated [116]. A CDC bioreactor was used to grow *S. mutans* biofilms on hydroxyapatite coupons, and then the biofilms were treated with silver nanoparticles in microtiter plates. The results show that a 2.3 log CFU reduction in *S. mutans* in biofilms was observed at a 100 ppm concentration, and up to 7 log reduction was achieved when the concentration was increased to 1000 ppm. In general, a thicker coating of antimicrobials should prevent biofilm formation. However, the thickness of multiple antimicrobial coatings is weakly correlated with their antimicrobial effects on *Staphylococcus capitis* biofilms, but this correlation is not seen for *Microbacterium lacticum* biofilms [117].

Essential oils have been addressed on a global scale for their antimicrobial effects [83,118,119]. Keelara et al. [83] reported the significant effects of cinnamaldehyde (Sigma-Aldrich, St. Louis, MO, USA) and Sporan^®^ (EcoSmart Tech, Alpharetta, GA. USA)against *Salmonella* in biofilms. The bactericidal effects of these essential oils increased with an increase in their concentration—a 6 log CFU reduction in *Salmonella* biofilm was observed at a 2000 ppm concentration. Lamarra et al. [120] examined the antimicrobial effects of the controlled release of cabreuva (CE) essential oils embedded in a Polyvinyl alcohol (PVA) electrospun matrix. This release-based antimicrobial treatment was effective against *Candida albicans*, *E. coli*, *S. aureus*, and *S. epidermidis*. The inhibition zones of *S. aureus* were significantly higher (2.1 cm) compared to those of *E. coli* (1.6 cm), indicating that the antimicrobial capacity of PVA + CE against Gram-positive bacteria was higher than that against Gram-negative bacteria.

A relatively newer and more cutting-edge technology involves the use of superhydrophobic surfaces for biofilm mitigation. Unlike release-based and contact-based antimicrobial coatings, these surfaces possess a water-repellent quality, which prohibits bacterial attachment to the surface, thereby preventing biofilm formation [115]. Bruzaud et al. [121] evaluated the efficacy of superhydrophobic steel via the electrodeposition of hydrophobic polymers, and found that *P. aeruginosa* was significantly reduced by 3 log when superhydrophobic surfaces were used, with smaller water sliding contact angles compared to other surfaces. Further, *L. monocytogenes* exhibited stronger anti-biofilm (2.9 log reduction) effects when superhydrophobic surfaces with a lower water sliding contact angle were employed.

While these coatings exhibit promising results in the control and prevention of biofilm formation, the comprehensive understanding and strategic management of their limitations are essential to the advancement of effective biofilm management and prevention methodologies. Some of the drawbacks related to contact-based coatings may include effectiveness over time, environmental impact, pre-existing surface irregularities, the impacts of the coatings on consumers or users (especially for food and medical products) and cost. Release-based coatings also show drawbacks, such as antimicrobial resistance, the depletion of antimicrobial effect over-time and environmental impact. Superhydrophobic surfaces and repulsion-based techniques have their share of drawbacks as well—they are expensive, and they only represent a preventative measure and do not kill the bacteria, unlike contact- and release-based techniques.

## 4. Conclusions

Multispecies bacterial communities are prevalent in natural environments, and the utilization of bioreactors for their assessment is a vital area of research with direct relevance to diverse fields, including food science, environmental science, medicine, biotechnology, and space science, among others. Specifically, the prevalence of biofilms in food industry settings, such as on food contact surfaces, underscores the significance of understanding biofilm formation. Since there are a multitude of factors responsible for biofilm formation, such as surface properties, shear stresses, environmental conditions and bacterial characteristics, to name a few, the investigation of biofilm-related phenomena is quite complex. Further, this review emphasizes that the process of biofilm formation is not only shear-dependent, but also depends on the hydrodynamic conditions within the bioreactors employed to examine the impact of the applied shear stress. Besides investigating biofilm formation, studies have shown that there is a vital link between the shear stress involved in biofilm formation and detachment. The judicious selection of bioreactors is extremely critical for studying biofilm growth and removal, and for developing preventative and control measures. While investigating biofilm removal techniques, especially from the perspective of food safety, prioritizing preventative strategies is crucial. Besides sanitary design and operation practices, the development of antimicrobial coatings used as sustainable biofilm-removal treatments is imperative, and these attempts must adopt a holistic perspective when addressing the challenges associated with biofilms.

## 5. Disclaimer

USDA is an equal opportunity provider and employer.

## Figures and Tables

**Table 1:** Social behavior of bacteria in biofilms when co-inoculated with other bacteria.

Social Behavior	Species	Effects	References
Cooperative interaction	*Listeria monocytogenes* and *Salmonella Typhimurium*	Metabolic collaboration during biofilm formation	[17]
Competitive interaction	*L. monocytogenes* and *Bacillus cereus*	Restrained *L. monocytogenes* growth and biofilm formation by *Bacillus cereus*	[18]
Competitive interaction	*P. putida* strains and *Salmonella java*	Mutual inhibition, potential use of *P. putida* as biocontrol agents against *S. java*	[19]
Competitive interaction	*Escherichia coli*, *Vibrio cholerae*, *Bdellovibrio bacteriovorus*	Predation—*B. bacteriovorus* is predator whereas *E. coli* and *V. cholerae* are prey	[20]
Cooperative interaction	*P. aeruginosa* and *Staphylococcus aureus*	Mutual defense and metabolic cooperation against antibiotics from these cystic fibrosis-adapted strains	[21]
Competitive interaction	*Salmonella Typhimurium* wild type and mutant with *E. coli*	Outgrowth of *Salmonella* strains and suppression of matrix production by *E. coli* within the biofilm	[22]
Cooperative interaction	*Streptococcus oralis*, *Actinomyces oris*, *Candida albicans*	Promotion of biofilms and planktonic environments among all three species	[23]
Competitive interaction	probiotic *E. coli*, shiga-toxigenic *E. coli*, *P. aeruginosa*, *S. aureus*, and *Staphylococcus epidermidis*	Suppression of *E. coli* as well as *S. aureus* and *S. epidermidis* biofilms by probiotic *E. coli* strain	[24]

**Table 2:** Fluid flow conditions and flow parameters used in biofilm evaluation.

Flow Conditions	Flow System	Bacteria	Flow Parameters	Results	References
Stagnant and shaken fluid, laminar	Flow chamber	*Pseudomonas fluorescens*	Shear stress: 1.39 × 10^−4^ and 8.33 × 10^−4^ Pa for laminar flow	Clumps in biofilm under shaking fluid conditions; higher shear stress promotes EPS formation and dense biofilms	[43]
Laminar and turbulent	Closed-loop system	Seawater bacterial consortium	Flow rates: laminar—0.023 m/s, turbulent—0.052 m/s	Highly prevalent bio-corrosion on weld joints under laminar flow	[44]
Laminar	Flow chamber	*Shewanella oneidensis*	Flow rate—0.1 to 0.8 mL/min (equivalent shear stress: 2 to 16 mPa)	Higher rate of biofilm removal at higher flow rates	[45]
Static, laminar, and turbulent	Parallel flow cell system	*Bacillus* sp.	Shear stress of 0.23, 0.68, 1.39, 2.30 Pa	Complexly structured biofilms at lower shear stress; biofilms with dense and smooth structures and higher adhesive strength under turbulent flow	[46]
Laminar	Microfluidic device	*E. coli* and *S. aureus*	Flow rates: 0.015, 0.03, 0.04, and 0.05 mL/min	Higher shear force required to inhibit biofilm formations on hydrophilic surfaces	[47]
Laminar	PDMS microcha-nnels	*S. aureus*	Shear stress: 0.015 to 0.15 Pa	Tower-like structures formed during biofilm formation at ~0.06 Pa shear stress	[48]
Turbulent	Rotating cylinder reactor	*Bacillus cereus* and *P. fluorescens*	Shear stress: 0.70, 1.66,5.50, 10.9, 17.7 Pa	Higher rate of biofilm removal under low shear stress	[49]
Laminar and Turbulent	Microfluidic flow channel	*S. mutans*, *S. epidermidis*, *P. aeruginosa*	Shear stress: 0.6, 4.1, 11.5, 23.8, 35.5, 55.3 Pa	Source of microspray affects crescent or curved shapes of ripples in biofilms; wrinkled surface with *P. aeruginosa* biofilm	[50]
NA	Rotating disc system	*Candida albicans*	Shear stress: 0.003, 0.110, 0.198 Pa	Shear stress and growth phases affect biofilm formation	[51]
Turbulent	Simulated cooling water system	*P. fluorescens*	Flow rate: 0.6,1.0,1.6 m/s	Adhesive strength of biofilms increases with flow rate	[52]

## Data Availability

Data are contained within the article.
